# Prehypertension Is Not Associated with All-Cause Mortality: A Systematic Review and Meta-Analysis of Prospective Studies

**DOI:** 10.1371/journal.pone.0061796

**Published:** 2013-04-25

**Authors:** Xiaofan Guo, Xiaoyu Zhang, Liqiang Zheng, Liang Guo, Zhao Li, Shasha Yu, Hongmei Yang, Xinghu Zhou, Lu Zou, Xingang Zhang, Zhaoqing Sun, Jue Li, Yingxian Sun

**Affiliations:** 1 Department of Cardiology, The First Hospital of China Medical University, Shenyang, People’s Republic of China; 2 Shenyang Eye Research Institute, The Fourth People’s Hospital, Shenyang, People’s Republic of China; 3 Department of Clinical Epidemiology, Library, Shengjing Hospital of China Medical University, Shenyang, People’s Republic of China; 4 Department of Cardiology, Shengjing Hospital of China Medical University, Shenyang, People’s Republic of China; 5 Heart, Lung and Blood Vessel Center, Tongji University, Shanghai, People’s Republic of China; University of British Columbia, Canada

## Abstract

**Objectives:**

Quantitative associations between prehypertension or its two separate blood pressure (BP) ranges and cardiovascular disease (CVD) or all-cause mortality have not been reliably documented. In this study, we performed a comprehensive systematic review and meta-analysis to assess these relationships from prospective cohort studies.

**Methods:**

We conducted a comprehensive search of PubMed (1966-June 2012) and the Cochrane Library (1988-June 2012) without language restrictions. This was supplemented by review of the references in the included studies and relevant reviews identified in the search. Prospective studies were included if they reported multivariate-adjusted relative risks (RRs) and corresponding 95% confidence intervals (CIs) of CVD or all-cause mortality with respect to prehypertension or its two BP ranges (low range: 120–129/80–84 mmHg; high range: 130–139/85–89 mmHg) at baseline. Pooled RRs were estimated using a random-effects model or a fixed-effects model depending on the between-study heterogeneity.

**Results:**

Thirteen studies met our inclusion criteria, with 870,678 participants. Prehypertension was not associated with an increased risk of all-cause mortality either in the whole prehypertension group (RR: 1.03; 95% CI: 0.91 to 1.15, *P = *0.667) or in its two separate BP ranges (low-range: RR: 0.91; 95% CI: 0.81 to 1.02, *P = *0.107; high range: RR: 1.00; 95% CI: 0.95 to 1.06, *P* = 0.951). Prehypertension was significantly associated with a greater risk of CVD mortality (RR: 1.32; 95% CI: 1.16 to 1.50, *P*<0.001). When analyzed separately by two BP ranges, only high range prehypertension was related to an increased risk of CVD mortality (low-range: RR: 1.10; 95% CI: 0.92 to 1.30, *P* = 0.287; high range: RR: 1.26; 95% CI: 1.13 to 1.41, *P*<0.001).

**Conclusions:**

From the best available prospective data, prehypertension was not associated with all-cause mortality. More high quality cohort studies stratified by BP range are needed.

## Introduction

High blood pressure (BP) is the leading cause of disease burden worldwide [1]. Suboptimal BP is responsible for a huge economic and health burden in both developed and developing countries [2]. Worldwide, more than seven million premature deaths can be attributed directly or indirectly to hypertension [3]. Complications of hypertension affect life quality substantially because many crucial organs, such as heart, brain and kidney, are involved and damaged. It has become an important public-health challenge to the world since the number of hypertensive people is extremely large [4].

The association between high BP and cardiovascular disease (CVD) and mortality is well established [5]–[7]. BP is strongly related to vascular mortality, down to at least 115/75 mm Hg [5]. The seventh report of the Joint National Committee on Prevention, Detection, Evaluation, and Treatment of High Blood Pressure (JNC 7) timely updated the BP category, coming up with the concept of prehypertension for better management [8]. Numerous studies emerged afterward to investigate the risk of prehypertension for various types of adverse outcomes, including stroke, coronary heart disease, and CVD and all-cause mortality [9]–[13].

Risk of mortality provides evidence for the prevention and treatment strategies of prehypertension. Since the ultimate public health goal of antihypertensive therapy is to reduce cardiovascular or total mortality, it is important to recognize first how risky prehypertension is for CVD or total death. Although a few studies pertaining to this issue exist, the conclusion has been compromised by the inconsistent results. It is difficult to assess this issue in a single study due to limited events. To our knowledge, there has been no quantitative analysis conducted to investigate the relationship between prehypertension and CVD or all-cause mortality from the literature worldwide. Therefore, we performed this meta-analysis to characterize the magnitude of these associations on a prospective level.

## Materials and Methods

### Literature Search

We performed and reported a systematic review of the published literature according to the recommendations of the Meta-analysis of Observational Studies in Epidemiology Group [14] and the PRISMA (Preferred Reporting Items for Systematic Reviews and Meta-Analysis) Statement [15]. We conducted a comprehensive search of PubMed (1966-June 2012) and the Cochrane Library (1988-June 2012) without language restrictions. Search terms including MeSH words and text words were related to exposure (“prehypertensi*” or "high normal blood pressure") and to outcomes (“mortality”, “survival”, “death” or “fatal”). Two authors (Guo X and Zhang XY) independently screened the studies fulfilling the inclusion criteria. In addition, we manually searched the references in the articles chosen for data abstraction and in the relevant reviews identified in the search. If the articles did not contain all of the necessary information, we contacted the authors for any possible additional published or unpublished data.

### Inclusion and Exclusion Criteria

For inclusion, studies had to meet the following criteria: (1) original article, prospective cohort design; (2) assessment of prehypertension or high normal BP as baseline exposure; (3) assessment of CVD mortality or all-cause mortality as outcome; (4) follow-up of at least 5 years, and (5) reported association measures [relative risk (RR) or hazard ratio (HR) and 95% confidence interval (95% CI)] from the multivariate-adjusted analyses between exposure and outcomes with normal BP as reference. Multiple samples with different gender, age or ethnic groups based on the same population were also included. If multiple reports from the same study were identified, we used the one with the most detailed information and supplemented it.

Studies were excluded if they met the following criteria: (1) no original data (reviews, comments); (2) only unadjusted or age- and gender-adjusted RR or HR was reported; (3) duplicated studies; (4) not conducted in human, and (5) data were derived from secondary analysis of clinical trials.

### Data Extraction

Using a standardized data extraction form, two investigators (Guo L and Li Z) extracted the data independently with discrepancies resolved by an additional reviewer (Zheng L) and through discussion. Information extracted included first author’s name, publication year, country, sample characteristics, prevalence of prehypertension, follow-up, definition of high BP, adjusted variables, outcome assessment, and multivariate-adjusted RRs or HRs and corresponding 95% CIs. An electronic abstraction database was created in Microsoft Excel.

### Assessment of Study Quality

According to the guidelines developed by the US Preventive Task Force and the modified checklist used in previous studies [16]–[18], we assessed quality of all articles that met the selection criteria with the following eight items: (1) prospective study design; (2) maintenance of comparable groups; (3) adjustment of potential confounders; (4) documented loss of follow-up rate; (5) outcome assessed blind to exposure status; (6) clear and proper definition of exposures (prehypertension) and outcomes (CVD and all-cause mortality); (7) temporality (BP measured at baseline, not at time of outcomes assessment) and (8) follow-up of at least one year. Studies were graded as of good quality if they met 7 to 8 criteria; fair if they met 4 to 6; and poor if they met fewer than 4 criteria.

### Statistical Analyses

Prehypertension was defined as systolic blood pressure (SBP) at 120–139 mmHg or diastolic blood pressure (DBP) at 80–89 mmHg, with two BP ranges further divided (i.e. low range: SBP 120–129 mmHg or DBP 80–84 mmHg and high range: SBP 130–139 mmHg or DBP 85–89 mmHg). Normal BP (SBP<120 mmHg and DBP<80 mmHg) was taken as the reference category for RRs. Outcome assessment was the relative risk of CVD and all-cause mortality in the low-range and high-range prehypertension categories, respectively or in the whole prehypertensive range.

To estimate quantitative associations between prehypertension and the mortality outcomes, we obtained pooled estimates basing on the multivariate-adjusted RRs or HRs with 95% CIs from included studies. Between-study heterogeneity was evaluated by Q-statistic and quantified by the I^2^ statistic. I^2^ statistic of 0%–40% indicates unimportant heterogeneity, 30%–60% indicates moderate heterogeneity, 50%–90% indicates substantial heterogeneity, and 75%–100% indicates considerable heterogeneity [19]. If statistically significant heterogeneity was considered present (P<0.1 and I^2^>50%), we chose a random-effects model, otherwise, a fixed-effects model was used. Subgroup analyses were performed to explore the heterogeneity according to average age (<65 years vs. ≥65 years), gender (men vs. women), location (Asian vs. non-Asian), sample size (<10000 vs. ≥10000), follow-up (<10 years vs. ≥10 years) and study quality (good vs. fair). Possible publication bias was evaluated visually using funnel plots and statistically by Begg’s and Egger’s tests. Sensitivity analysis was used to examine the influence of individual studies to see the extent to which inferences depend on a particular study or group of studies. All analyses were performed using the statistical package Stata version 11.0.

## Results

### Search Results

**Figure 1:**
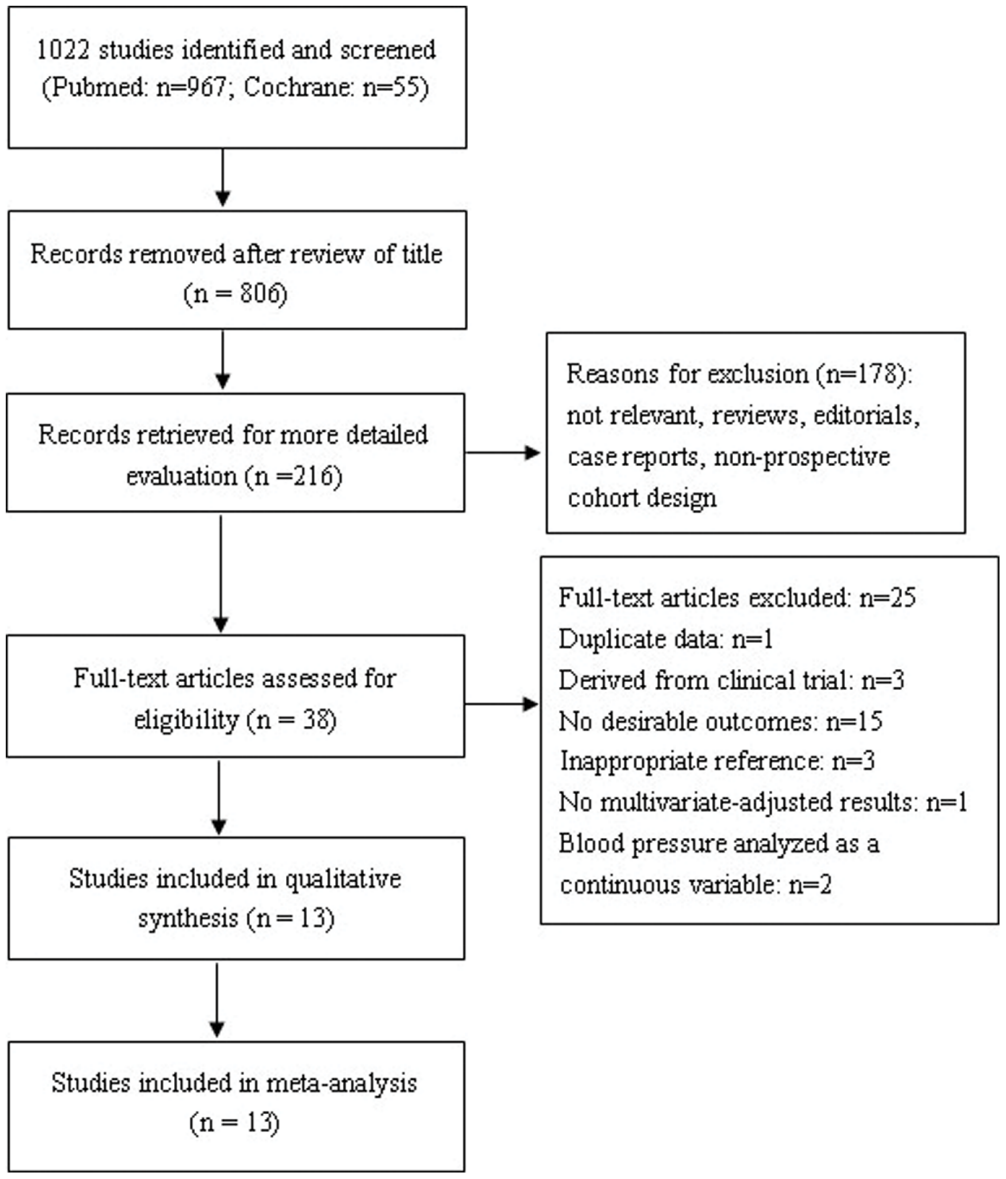
Flow chart of the study selection process.

A total of 1022 papers were identified from the initial database search, of which 984 were excluded following review of the title and abstract. The large majority of articles were excluded because they were not relevant to the issue we aimed to evaluate. Among the retrieved 38 articles, 13 cohort studies met our inclusion criteria, with 870,678 participants [20]–[32]. Figure 1 provides a diagram of the selection process and reasons for exclusion.

### Study Characteristics

Table 1 summarizes the characteristics of the individual studies. The included studies varied in sample size from 2376 [31] to 347,978 [21]. All but three of the studies [21], [22], [28] included both men and women. Follow-up ranged from 5 to 25 years. Five of the studies were conducted in the United States, two each in China, Korea and Japan, and one each in India and Singapore. We restricted the inclusion criteria to prospective studies with at least a minimum of 5 years follow-up to ensure a reliable conclusion. Eight of the included articles had good study quality.

**Table 1:** Description of characteristics from prospective studies included in the systematic review and meta-analysis.

First author, Publication year	Study	Country	Prevalence (pre-HTN)	Sample size (% men)	Follow-up (y)	Age, y (mean, range or SD)	Definition of pre-HTN	Adjusted variables	Studyquality
Arch G. Mainous III,2004 [^20^]	NHANES II, merged with the NH2MS	United States	28.7%	9087(NA)	12	30–74	JNC 7	Age, race, sex, smoking, BMI, exercise,total cholesterol, DM, heartfailure, heart attack and stroke	Fair
Paul D. Terry, 2006 [^21^]	MRFIT	United States	NA	347978(100)	25	35–57	JNC 7	Age, race/ethnicity, income, serum cholesterol level, smoking and use of medication for DM	Fair
Judith Hsia, 2007 [^22^]	WHI	United States	38.8%	60785(0)	7.7	62.8 (7)	JNC 7 or JNC 6	Age, BMI, DM, high cholesterol and smoking	Good
Qiuping Gu, 2008 [^23^]	NHANES III mortality study	United States	30.8%	16917(42)	8.5	≥18	JNC 7	Age, sex, race/ethnicity, leisure timephysical activity, smoking, obesity,hypercholesterolemia, DM, chronic kidney disease, and a history of congestiveheart failure, heart attack or stroke	Good
Jeannette Lee, 2008 [^24^]	Singapore CardiovascularCohort Study	Singapore	28.5%	5830(49)	12	39.8 (12.9)for pre-HTN	JNC 7	Age, sex, BMI, ethnic group, total-cholesterol/HDL-cholesterol, study, DM, CVD, smoking and alcohol intake	Good
Dongfeng Gu, 2009 [^25^]	China National HypertensionSurvey	China	34.5%	158666(49)	7.8	56 (≥40)	JNC 7	Age, sex, high school education,smoking, alcohol consumption, physical activity,BMI, antihypertensive medication, history ofCVD or DM, geographic region andurbanization	Good
Ai Ikeda, 2009 [^26^]	JPHC Study	Japan	43.0%	33372(35)	11	54 (40–69)	2003 European guidelines	Age, BMI, smoking, ethanol intake, antihypertensive medication, DM, serum total cholesterol levels and public health center areas	Good
Mangesh S. Pednekar,2009 [^27^]	Mumbai cohort	India	38.8%	148173(59)	5.5	50 (≥35)	JNC 7	Age, education, religion, mother tongue, tobacco habit and BMI	Fair
Tsogzolmaa Dorjgochoo, 2009 [^28^]	Shanghai Women’s Health Study	China	39.0%	68438 (0)	5	55 (40–70)	JNC 7 or 2007 Europeanguidelines	Education, waist-to-hip ratio, smoking, history of CVD and DM	Fair
Carlos Lorenzo, 2009 [^29^]	San Antonio Heart Study	United States	31.6%	3632 for all cause, 3580 for CVD mortality	15.2	25–64	JNC 7 or JNC 6	Age, sex, ethnicity, education, BMI, smoking and total cholesterol concentration	Good
Atsushi Hozawa, 2009 [^30^]	Ohsaki Cohort Study	Japan	41.8%	12928(43)	11.7	61.2 (9.4)	JNC 7	Age, sex, smoking, hyperglycemia, total cholesterol and BMI	Good
Nan Hee Kim, 2011 [^31^]	SWS Study	Korea	28.7%	2376(22)	7.6	>60	JNC 7 or 2007 Europeanguidelines	Age, sex, BMI, fasting glucose, HDL, total cholesterol and smoking	Good
Bayasgalan Gombojav, 2011 [^32^]	The Kangwha Cohort Study	Korea	NA	2496(42.3)	11.8	64–101	JNC 7	Age, education, smoking, drinking, activities of daily living, instrumental activities of daily living, chronic disease and antihypertensive therapy	Good

Pre-HTN: prehypertension; NA: not available; SD:standard deviation; BMI: body mass index; DM: diabetes mellitus; CVD: cardiovascular disease; HDL: high density lipoprotein. JNC 7: prehypertension (120–139/80 –89 mmHg); JNC 6, 2003 European guidelines and 2007 European guidelines: high normal blood pressure (130–139/85–89 mmHg) and normal blood pressure (120–129/80–84 mmHg).

### Prehypertension and All-cause Mortality

**Figure 2:**
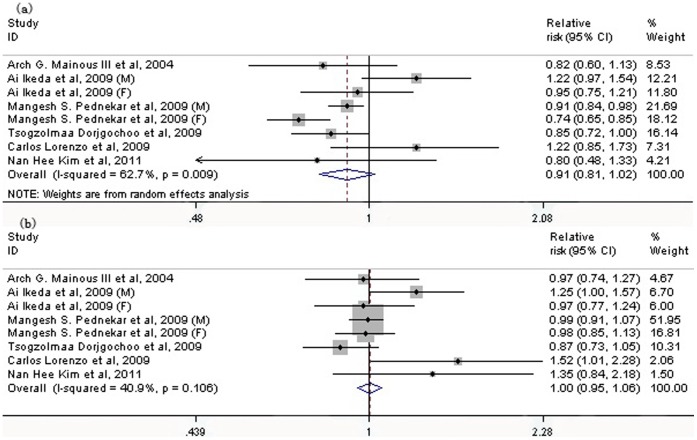
Association between two ranges of prehypertension, low range (a) and high range (b), and the risk of all-cause mortality. Low range prehypertension: 120–129/80–84 mmHg; high range prehypertension: 130–139/85–89 mmHg. CI: confidence interval.

**Figure 3:**
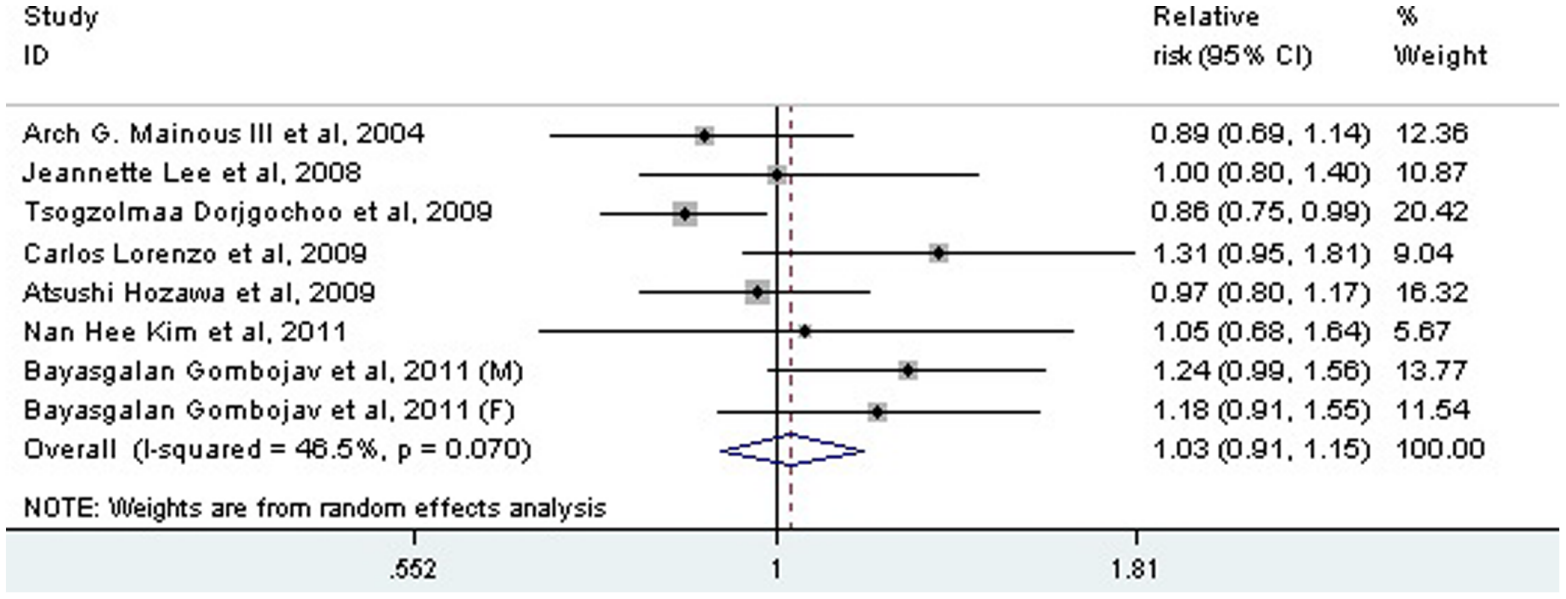
Association between prehypertension and the risk of all-cause mortality. CI: confidence interval.

In the pooled analysis from 8 populations, both low-range and high-range prehypertension were not associated with a greater risk of all-cause mortality (low-range: RR: 0.91; 95% CI: 0.81 to 1.02, *P* = 0.107; high range: RR: 1.00; 95% CI: 0.95 to 1.06, *P* = 0.951) (Figure 2a and b). Seven studies investigated the association between the whole range of prehypertension and all-cause mortality, the pooled result of which showed that prehypertension was not related to a greater risk of all-cause mortality (RR: 1.03; 95% CI: 0.91 to 1.15, *P* = 0.667), with some heterogeneity between studies (I^2^ = 46.5%, *P* = 0.07) (Figure 3).

### Prehypertension and Cardiovascular Disease Mortality

**Figure 4:**
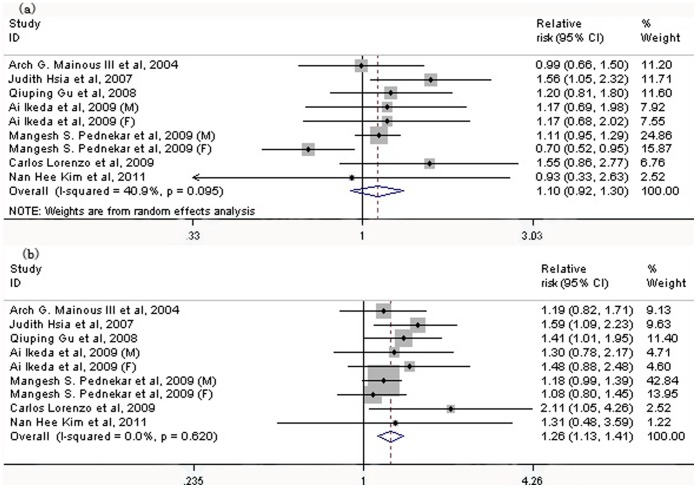
Association between two ranges of prehypertension, low range (a) and high range (b), and the risk of CVD mortality. Low range prehypertension: 120–129/80–84 mmHg; high range prehypertension: 130–139/85–89 mmHg. CI: confidence interval; CVD: cardiovascular disease.

**Figure 5:**
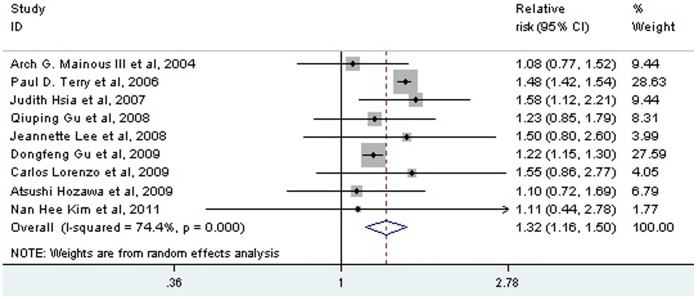
Association between prehypertension and the risk of CVD mortality. CI: confidence interval; CVD: cardiovascular disease.

Seven studies with nine populations evaluated the risk of low-range and high-range prehypertension for CVD mortality separately. Only high range prehypertension was associated with an increased risk of CVD mortality (low-range: RR: 1.10; 95% CI: 0.92 to 1.30, *P* = 0.287; high range: RR: 1.26; 95% CI: 1.13 to 1.41, *P*<0.001) (Figure 4a and b). Among the whole range prehypertensive populations, the risk of CVD mortality was also increased (RR: 1.32; 95% CI: 1.16 to 1.50, *P*<0.001) (Figure 5), with some heterogeneity between studies (I^2^ = 74.4%, *P*<0.001).

### Sources of Heterogeneity

Table 2 shows the further analyses stratified by various population groups in each range of prehypertension. The heterogeneity of effect was due to differences in gender, age, follow-up or study quality. No publication bias was observed (Begg’s test all *P*>0.05; Egger’s test all *P*>0.05, figures not shown). The sensitivity analysis showed that the omission of any of the studies from the analysis did not alter the overall finding.

**Table 2:** Subgroup analyses to explore source of heterogeneity.

Subgroups	CVD mortality			All-cause mortality		
	No.	RR (95%CI),		No.	RR (95%CI),	
		*P* for heterogeneity			*P* for heterogeneity	
**Low range (SBP 120–129 mmHg or DBP 80–84 mmHg)**						
**Gender**						
Men	3	1.11 (0.97–1.28)		2	1.03 (0.78–1.37)	
Women	4	1.13 (0.72–1.75)		3	0.822 (0.72–1)	
		0.46			0.014	
**Age group**						
≥65 y	3	0.82 (0.57–1.19)		1	0.8 (0.48–1.33)	
<65 y	8	1.14 (0.93–1.41)		7	0.92 (0.81–1.03)	
		0.146			0.679	
**Location**						
Asian	5	0.99 (0.77–1.26)		6	0.9 (0.79–1.02)	
Non-Asian	4	1.27 (1.03–1.58		2	0.99 (0.67–1.46)	
		0.09			0.424	
**Sample size**						
<10000	3	1.13 (0.82–1.55)		3	0.94 (0.71–1.24)	
≥10000	6	1.09 (0.88–1.36)		5	0.9 (0.79–1.03)	
		0.811			0.582	
** Follow-up**						
<10 y	5	1.07 (0.82–1.4)		4	0.84 (0.74–0.94)	
≥10 y	4	1.16 (0.9–1.49)		4	1.04 (0.86–1.25)	
		0.573			0.011	
** Study quality**						
Good	6	1.31 (1.06–1.61)		4	1.07 (0.9–1.27)	
Fair	3	0.93 (0.69–1.26)		4	0.84 (0.75–0.94)	
		0.037			0.005	
**High range (SBP 130–139 mmHg or DBP 85–89 mmHg)**						
** Gender**						
Men	3	1.2 (1.04–1.4)		2	1.02 (0.94–1.1)	
Women	4	1.33 (1.09–1.62)		3	0.94 (0.85–1.04)	
		0.443			0.242	
** Age group**						
≥65 y	3	0.84 (0.59–1.21)		1	1.35 (0.84–2.18)	
<65 y	8	1.27 (1.13–1.43)		7	0.997 (0.94–1.06)	
		0.034			0.217	
** Location**						
Asian	5	1.19 (1.04–1.36)		6	0.99 (0.94–1.06)	
Non-Asian	4	1.44 (1.18–1.75)		2	1.11 (0.89–1.39)	
		0.113			0.344	
** Sample size**						
<10000	3	1.34 (0.99–1.83)		3	1.15 (0.94–1.41)	
>10000	6	1.25 (1.11–1.41)		5	0.99 (0.93–1.05)	
		0.678			0.159	
** Follow-up**						
<10 y	5	1.24 (1.09–1.4)		4	0.98 (0.92–1.04)	
>10 y	4	1.36 (1.07–1.74)		4	1.11 (0.97–1.27)	
		0.486			0.09	
** Study quality**						
Good	6	1.49 (1.23–1.8)		4	1.18 (1.02–1.36)	
Fair	3	1.16 (1.01–1.33)		4	0.97 (0.91–1.04)	
		0.035			0.018	

CVD: cardiovascular disease; RR: relative risk; CI: confidence interval;

SBP: systolic blood pressure; DBP: diastolic blood pressure.

## Discussion

The present study provided for the first time a comprehensive review of the literature and quantitative estimates of prospective associations between prehypertension and CVD and all-cause mortality. We found that prehypertension, including both ranges, was not associated with all-cause mortality. The positive association between prehypertension and the risk of CVD mortality was confined to the high range BP group when analyzed by two ranges separately. The effects of prehypertension on mortality differed by many factors, such as age group, sample size and study quality.

A high prevalence of prehypertension was observed in many areas of the world [33]–[36]. In the United States, the overall prevalence of prehypertension was approximately 31% according to the Third National Health and Nutrition Examination Survey (NHANES III) [37], and 3 of 8 adults had low range prehypertension and 1 of 8 adults had high range prehypertension from 2005 to 2006 [38]. Considering this large population and the high progression rate from prehypertension to hypertension, the burden is large.

A previous meta-analysis of 61 prospective studies indicated that the risk of cardiovascular mortality began to increase from BP values of 115/75 mmHg, and doubled for each 20 mmHg rise in SBP and 10 mmHg rise in DBP among 40–69-year-olds [5], suggesting that a BP range of 115–140/75–90 mmHg might also cause adverse outcomes and merits attention. In the present study, we quantitatively estimated that the risk of CVD mortality was increased ∼1.3-fold in the prehypertensive range compared to normal BP, the risk ratio of which was lower than the result by Lewington et al [5]. This might be explained by the various age groups included. When the data were analyzed by two BP ranges separately, we found that only high range prehypertension was related to a greater risk of CVD mortality. Although the relatively small number of deaths in the low range BP group is an alternative explanation, this is in accordance with the higher risk of CVD morbidity or mortality in the BP range of 130–139/85–89 mmHg described in previous studies [39]–[41], underscoring the differences between the two BP ranges of prehypertension.

Interestingly, we found that there was no relationship between prehypertension or either of its two ranges and all-cause mortality. Although a previous pooled study of Japanese subjects indicated that prehypertension was significantly associated with all-cause mortality following multivariate-adjustment, the positive result was mild and limited to only two decades of age [42]. There might exists an age- or gender-specific relationship which our study failed to reach, but it seems plausible that there is no real association between prehypertension and all-cause mortality.

We assumed that the inconsistent results between CVD mortality and all-cause mortality we observed in the present study might be caused by the different data set included in the two analyzing groups. Previous studies showed that the relation of BP to CVD mortality or all-cause mortality was closely associated with age [5], [42]. Although the studies we included were all age-adjusted, the different age distributions in the two analyzing data set may still have an effect on the outcomes. In addition, there were many other causes of mortality, such as cancer or accidental death, which might decrease in proportion to CVD mortality. For example, Terry et al reported that the rate of all external death was 4.95% in normal BP group, compared to a lower one of 4.47% in prehypertension group [21].

Lifestyle modifications have been shown to effectively lower BP and are regarded as the first choice for prehypertension management. Although a few studies have demonstrated good efficacy and safety of antihypertensive treatment for prehypertension [43]–[45], it is not known whether the benefits of pharmacological therapy outweigh the harms. Our data provide evidence for the establishment of prevention and treatment strategies of prehypertension. Different effects of two BP ranges in prehypertension on future outcomes should be fully considered.

There are limitations in the present study that merit discussion. First, the contributing studies varied in their initial exclusion criteria and their inclusion of adjustment confounders. Although we only included multivariate-adjusted studies to minimize the impact, it remains a possibility that residual confounding across the studies caused overestimation of the associations. Second, since individual patient data or original data were unavailable, our intent to do more detailed relevant analyses, such as analysis by each decade of age, was restricted. In addition, a delay between search and publication was inevitable. Although our literature search was extensive, there still was a possibility of omission.

## Conclusions

From the best available prospective data, prehypertension was not associated with all-cause mortality. More high quality cohort studies stratified by BP range are needed. Different effects of two ranges in prehypertension on future outcomes should be fully considered when making prevention and treatment strategies.
